# Self-Reported Chronotype and Diet Quality in Chinese Adolescents: Insights from a Cross-Sectional Study in Xiamen

**DOI:** 10.3390/nu18183097

**Published:** 2026-09-21

**Authors:** Hongge Tang, Xiangquan Liu, Zhiyong Zou, Qi Sun

**Affiliations:** 1 School of Public Health and Medical Technology, Xiamen Medical College, Xiamen 361023, China; thg@xmmc.edu.cn (H.T.); lxq@xmmc.edu.cn (X.L.); 2 Institute of Child and Adolescent Health, School of Public Health, Peking University, National Health Commission Key Laboratory of Reproductive Health, Beijing 100191, China; 3 School of Public Health, China Medical University, Shenyang 110013, China

**Keywords:** sleep chronotype, dietary quality, circadian rhythm, adolescents, Global Diet Recommendations

## Abstract

Background: External constraints can disrupt adolescents’ circadian rhythms, potentially increasing their risk of obesity and metabolic diseases. Methods: This cross-sectional study examined the association between chronotype and dietary quality among 982 adolescents recruited through stratified random sampling in Xiamen, China. Chronotype and dietary quality were assessed using the Morningness–Eveningness Questionnaire (MEQ-19) and the Diet Quality Questionnaire (DQQ), respectively. Results: Univariate analysis indicated that Global Diet Recommendations (GDR) scores, the index of DQQ, varied significantly across grades, BMI and chronotypes (*p* < 0.05). Multiple linear regression revealed a positive association between MEQ score and GDR-Total (*p* < 0.001), which appeared to be primarily related to GDR-Health rather than GDR-Limit. Sensitivity analyses confirmed the robustness of these associations when MEQ was treated as either a continuous or categorical variable. Exploratory restricted cubic spline analyses suggested an apparent inverted-U-shaped association between MEQ score and GDR-Total, although its shape was sensitive to knot specification and the inclusion of observations at the distributional tails. GDR-Health showed a predominantly increasing pattern, whereas GDR-Limit showed a U-shaped pattern. Conclusions: Thus, dietary quality initially improved as chronotype shifted from eveningness toward morningness but deteriorated at extreme morningness, suggesting an optimal chronotype range for healthy dietary patterns. These findings support personalized interventions for adolescents that combine improved sleep patterns with increased consumption of health-promoting foods, rather than focusing solely on restricting unhealthy foods.

## 1. Introduction

Obesity is a major global health challenge of the twenty-first century [1] and affects individuals across socioeconomic groups [2]. In 2022, the World Health Organization estimated that approximately 390 million children and adolescents aged 5–19 years were overweight, including 160 million with obesity [1]. Childhood obesity and its associated health risks may persist into adulthood [3]. During childhood and adolescence, adequate nutrition is essential for physical growth, cognitive performance, and social development, and may influence the long-term risk of non-communicable diseases [4,5,6]. Improving dietary quality during adolescence is therefore an important public health priority. School-based nutrition strategies, including plant-based dietary approaches, have also been proposed to improve adolescent dietary quality while supporting long-term sustainability [7].

Circadian rhythms are endogenous biological rhythms with a cycle of approximately 24 h [8,9]. Individual differences in the timing of sleep–wake and physiological processes are reflected in the chronotype, which is commonly classified as a morning, intermediate, or evening type [10,11,12]. Although chronotype is relatively stable during adulthood [13,14,15], adolescence is often accompanied by a shift toward eveningness due to biological maturation and psychosocial factors [16,17,18,19]. Compared with morning-type individuals, evening-type individuals are more likely to experience daytime sleepiness, insomnia, and poorer sleep quality [20,21,22,23]. Circadian disruption has also been associated with obesity and cardiometabolic diseases [24], while evidence from 24 h movement guidelines highlights the interconnected roles of sleep, physical activity, and sedentary behavior in adolescent health [25]. These findings have prompted interest in whether chronotype may also be associated with meal timing, dietary behaviors, food preferences, and energy metabolism [26].

This issue is particularly relevant among Chinese adolescents. A recent meta-analysis reported a high prevalence of sleep curtailment, sleep disorders, and delayed sleep phase in this population [27]. Chronotype distributions also vary across regions, with eveningness relatively common among high school students in Hong Kong [28], whereas the intermediate chronotype is predominant in cities such as Shenzhen and Taiyuan [29,30]. Dietary patterns among Chinese adolescents are also often characterized by high fat intake and insufficient consumption of fruits, vegetables, and other healthy foods [31,32]. Chronotype may be related to the timing of energy intake and food preferences [33]. Eveningness has been associated with a possible mismatch between biological timing and daily schedules [34] and with less favorable dietary choices, including greater reliance on processed and energy-dense foods [35]. Proposed explanations include alterations in hunger-related signals and food-related cognitive regulation, although these mechanisms have not been directly assessed in the present study [36]. However, the association between chronotype and multidimensional dietary quality among Chinese adolescents, as well as the specific form of this relationship, remains insufficiently characterized.

The Diet Quality Questionnaire (DQQ) used in this study was developed by Zou and colleagues within the framework of the Global Diet Quality Project. It was locally adapted using foods commonly consumed by Chinese children and adolescents and validated among individuals aged 7–18 years, providing a feasible tool for assessing previous-day dietary intake by food group [37]. This approach is consistent with the WHO-UNICEF technical consultation, which emphasized the need for harmonized, feasible, and low-burden tools for monitoring healthy diets across populations [38]. Unlike dietary assessment tools based primarily on internationally standardized food lists, the DQQ incorporates locally relevant foods and captures both food groups related to dietary adequacy and foods recommended to be limited [37]. Evidence from multinational analyses further suggests that locally adapted sentinel food lists can capture most foods consumed at the subnational level, although their coverage may depend on the inclusion of regionally important foods [39].

Xiamen provides a relevant regional setting for examining the relationship between chronotype and dietary quality, given its distinctive local lifestyle and dietary characteristics [40]. We therefore conducted a school-based cross-sectional survey among adolescents aged 13–19 years in a selected district of Xiamen City. Chronotype and dietary quality were assessed using the Morningness–Eveningness Questionnaire (MEQ-19) and the DQQ, respectively. We examined the associations of MEQ score with GDR-Total, GDR-Health, and GDR-Limit scores and evaluated the form of these associations after adjustment for age, gender, BMI, grade, household income, residence, paternal education, maternal education, and sleep duration. Given the cross-sectional design, this study was intended to characterize associations and generate hypotheses rather than establish causal relationships.

## 2. Methods and Materials

### 2.1. Study Participants

This study was conducted in one selected district of Xiamen City. Within this district, six schools were randomly selected. The student sampling frame was stratified by grade, including Grades 7–12, and eligible students were randomly selected within the participating schools and grade strata. The planned sampling target was 200 students per grade, corresponding to an intended total of 1200 students. However, 1017 questionnaires were ultimately distributed during the data-collection period.

Students aged 13–19 years who were able to independently understand and complete the questionnaires were eligible for inclusion. The exclusion criteria were severe cognitive impairment or a diagnosed psychiatric disorder, recent use of medications known to affect sleep or appetite, and absence of informed consent from a legal guardian. Of the 1017 questionnaires distributed, 982 participants were retained in the final analysis, corresponding to a final analytic retention rate of 96.6%.

Because the study was conducted in only one selected district, the sampling procedure should not be interpreted as providing a representative sample of all adolescents in Xiamen City or China. In addition, school- and class-level identifiers were not available in the de-identified dataset; therefore, potential clustering of students within schools or classes could not be formally accounted for using multilevel models or cluster-robust standard errors. This issue is acknowledged as a limitation of the present study.

Sample-size planning was informed by previous cross-sectional studies [41] and an a priori power analysis conducted using G Power version 3.1.9.7 (Heinrich Heine University Düsseldorf, Germany). The calculation assessed the incremental contribution of continuous MEQ score to GDR-Total after covariate adjustment. Assuming a medium incremental effect size of Cohen’s f^2^ = 0.15, α = 0.05, power = 0.95, one tested predictor, and 17 total predictor parameters excluding the intercept, the minimum required sample size was 90, with a corresponding power of 0.9520. The final analytic sample comprised 982 participants.

The study protocol, including the consent and assent procedures, was approved by the Ethics Committee of China Medical University (Approval No. [2024]059). Before completing the questionnaire, participants were informed about the study and the relevant consent or permission procedures were completed. For participants, parental or legal-guardian permission and adolescent assent were obtained before participation. Participation was voluntary, and participants could decline to participate or discontinue the questionnaire at any time before submission.

### 2.2. Research Instruments

A series of well-established and validated scales and tools were employed in this study to collect data across multiple dimensions, as detailed below:

#### 2.2.1. Sleep Chronotype Assessment

Sleep chronotype was assessed using the Chinese version of the MEQ-19, as culturally adapted and revised by Zhang et al. [42]. The MEQ-19 comprises 19 items addressing preferred bedtimes and wake-up times, diurnal activity preferences, and related factors. Total scores range from 16 to 86. Based on their total score, participants are classified into one of three chronotype categories: morning (59–86), intermediate (42–58), or evening type (16–41). Previous research has reported acceptable reliability and evaluated the psychometric properties of the Chinese version of the MEQ [42]. In the present sample, the questionnaire showed acceptable internal consistency, with a Cronbach’s α of 0.777.

#### 2.2.2. Dietary Quality Assessment

DQQ is a rapid dietary assessment tool that captures data at the food-group level and reflects dietary patterns through “sentinel foods”, which are defined as the foods consumed by more than 95% of individuals within each food group [43]. The Chinese version of the DQQ is based on 29 food groups and uses a “yes/no” response format for each group. Previous studies have validated its ability to capture dietary intake among Chinese children, adolescents, and adults [44,45]. The Chinese DQQ and additional information are available on the Global Diet Quality Project website [46]. Responses to the DQQ were used to calculate the GDR score, consisting of two core components as follows: GDR-Health score: includes nine groups of healthful foods (fruit and vegetables; legumes and pulses; nuts and seeds; whole grains and dietary fiber, etc.). One point is awarded for each group consumed, yielding a possible range of 0–9 points. This score reflects the participant’s intake of nutrient-rich foods. GDR-Limit score: includes eight groups of foods to be limited (total fat, saturated fat, added salt, free sugars, processed meats [2 points per group], unprocessed red meat, etc.). One point is awarded for each group consumed (processed meats count as two points), for a range of 0–9 points. This score reflects the intake of foods associated with poorer dietary quality. The overall GDR score is calculated as: GDR-Total score = GDR-Health score − GDR-Limit score, resulting in a range from −9 to +9, where a higher score indicates better overall diet quality.

The Chinese version of the DQQ has been evaluated in Chinese children and adolescents using the sentinel-food approach. This validation study showed that the DQQ captured more than 95% of participants consuming the assessed food groups, supporting its use for population-level diet-quality monitoring in Chinese children and adolescents [37]. The scores were categorized as follows: GDR-Health score: ≤1, =2, ≥3; GDR-Limit score: =0, =1, ≥2; GDR-Total score: <0, =0, ≥1 [37].

#### 2.2.3. Measurement of Other Variables

Demographic information was collected via a self-administered questionnaire, including gender, age, and grade level. Participants self-rated their household income relative to peers on a five-point scale: high, upper middle, middle, lower middle and low. Parental education was classified into four categories: junior high school or below, senior high school/vocational school, junior college, and bachelor’s degree or above. Sleep duration was assessed using an item from the Pittsburgh Sleep Quality Index (PSQI). Participants were asked, “During the past month, how many hours of actual sleep did you get at night?” The reported duration was categorized as ≥7 h or <7 h and included as a categorical covariate in the regression models. Anthropometric data were obtained through self-reported height (m) and weight (kg), from which BMI was calculated as weight divided by height squared (kg/m^2^). According to the “Screening Standard for Overweight and Obesity among Chinese Children and Adolescents” (WS/T586-2018) [47], BMI values were categorized based on exact age into normal weight, overweight, and obesity.

### 2.3. Quality Control

To ensure scientific rigor and data quality, quality-control procedures were implemented at multiple stages of the study. Each scale was administered in accordance with its standardized instructions to ensure consistent implementation. An expert panel assessed the content validity of the questionnaire instruments.

All survey administrators received standardized training in questionnaire administration, voluntary participation, consent and assent procedures, and quality-control requirements. Any issues identified during data collection were documented and addressed promptly. The approved consent and assent procedures were completed before questionnaire administration. Participation was voluntary, and participants could decline to participate or discontinue the questionnaire before submission.

All collected data were securely stored, and access was restricted to authorized members of the research team to protect confidentiality and privacy.

### 2.4. Data Collection and Processing

Data collection was carried out by trained and certified survey administrators. Electronic questionnaires were distributed online (via Wenjuanxing), using the prescribed stratified sampling procedure to ensure data authenticity and validity. Survey administrators explained the study purpose, key instructions, anonymization procedures, and the principle of voluntary participation to each student, ensuring informed and willing involvement. Administrators actively encouraged participation to meet the target sample size. Upon completion, 1017 questionnaires were returned.

For data processing, double data entry and cross-checking were performed in Excel to verify completeness. Questionnaires with missing data on MEQ and DQQ or logical inconsistencies (e.g., age not matching grade) were excluded to maintain accuracy and consistency and to minimize the impact of outliers. In total, 982 questionnaires were used for analysis in the present paper, a number that closely approximates the required sample size for this study.

### 2.5. Statistical Analysis

Statistical analyses were performed using IBM SPSS Statistics 21.0 and R version 4.6.0, as detailed below:

#### 2.5.1. Descriptive Analysis

Statistical analyses were conducted using IBM SPSS version 21.0. For continuous variables, data conforming to a normal distribution were summarized as mean ± standard deviation, whereas non-normally distributed variables were reported as median (M) with interquartile range (P25, P75). Categorical variables were expressed as frequencies and corresponding percentages. Descriptive statistics were systematically organized to generate tables illustrating the demographic and clinical characteristics of the study population, including distributions stratified by sleep chronotype, categorized GDR-Total score, and BMI classifications. These tables provided a comprehensive overview of the key variables and facilitated intuitive interpretation of population-level patterns.

#### 2.5.2. Univariate Analysis

Chi-square tests and non-parametric Kruskal–Wallis tests were employed to examine differences in demographic characteristics across sleep chronotype groups, GDR-Total score categories, and BMI classifications. The significance level was set at α = 0.05. Test results were interpreted in conjunction with the previously generated descriptive tables for sleep chronotype, GDR score grouping, and BMI stratification. Given the sample size of 982, the Kruskal–Wallis test statistic (H) was assumed to follow a chi-square (*χ*^2^) distribution under large-sample conditions. Accordingly, all Kruskal–Wallis test results were reported using the chi-square notation (*χ*^2^).

#### 2.5.3. Multiple Linear Regression

Associations between sleep chronotype (MEQ score) and GDR dimensions were examined using multiple linear regression models in R software (version 4.6.0). The MEQ score was treated as the independent variable, and GDR-Health, GDR-Limit, and GDR-Total scores were considered as dependent variables. Regression coefficients (*β*) and *p*-values were estimated to evaluate the strength and significance of the associations. Scatter plots with fitted regression lines and 95% confidence intervals were generated to visualize the relationships. The corresponding FDR-adjusted *p* values were calculated using the Benjamini–Hochberg procedure. Models were adjusted for age, gender, BMI, grade, household income, residence, paternal education, maternal education, and sleep duration.

#### 2.5.4. Sensitivity Analysis of the Association Between MEQ Scores and GDR Dimensions Using Multivariable Logistic Regression

To ensure the robustness of the association between MEQ scores and GDR dimensions, multivariable logistic regression models were employed for sensitivity analyses. In these models, MEQ scores were incorporated as both continuous and categorical variables (with the evening type as the reference group). After adjusting for covariates including age, BMI, gender, socioeconomic status, parental characteristics and sleep duration, we evaluated the associations between sleep chronotype and different GDR dimensions. Sleep duration, as a covariate, was categorized into two groups: ≥7 h and <7 h. In this study, adolescents with a sleep duration of ≥7 h accounted for 64.1% of the sample. The robustness of the observed associations between chronotype and dietary quality was assessed by examining the consistency of the odds ratios (ORs) across continuous and categorical MEQ specifications and prespecified GDR outcome contrasts. Standard ordinal logistic regression was not used because the proportional-odds assumption was not satisfied. Therefore, prespecified binary logistic contrasts were analyzed separately.

#### 2.5.5. Exploration of Nonlinear Relationships

Restricted cubic spline (RCS) analyses were conducted using R software version 4.6.0 and the rms package. For each GDR outcome, MEQ score was modeled using a restricted cubic spline with four prespecified knots located at the 5th, 35th, 65th, and 95th percentiles of the observed MEQ distribution. These knots corresponded to MEQ scores of 40, 48, 54, and 63, respectively. Percentile-based knots were selected to provide adequate flexibility across the observed MEQ range while limiting the influence of sparse observations at the distributional tails.

All models were adjusted for age, gender, BMI, grade, household income, residence, paternal education, maternal education, and sleep duration. Sleep duration was categorized as ≥7 h versus <7 h and entered as a categorical covariate. Adjusted predictions were calculated at the median age of 15.5 years, the median BMI of 20.2 kg/m^2^, the reference sleep-duration category of ≥7 h, and the modal categories of the other categorical covariates. MEQ scores were varied across the observed range to generate adjusted predicted GDR scores with 95% confidence intervals.

The RCS models were fitted using ordinary least-squares regression with the ols function in the rms package. Overall and nonlinear associations between MEQ score and each GDR outcome were evaluated using F tests implemented through the anova function. For the primary four-knot models, the overall association was assessed by jointly testing all three MEQ spline coefficients, whereas nonlinearity was assessed by jointly testing the two nonlinear spline coefficients, conditional on the linear MEQ term and the adjustment covariates. A *p* value < 0.05 for the nonlinear component was interpreted as evidence of departure from linearity. Because the RCS analyses were exploratory, these *p* values were interpreted as nominal.

To evaluate the robustness of the spline specification, sensitivity analyses were conducted using three and five knots. The three-knot model used knots at the 10th, 50th, and 90th percentiles, corresponding to MEQ scores of 42, 51, and 60. The five-knot model used knots at the 5th, 27.5th, 50th, 72.5th, and 95th percentiles, corresponding to MEQ scores of 40, 47, 51, 55, and 63. In addition, 1000 nonparametric bootstrap resamples and a tail-exclusion analysis excluding participants below the 5th or above the 95th MEQ percentiles were performed. All primary and sensitivity analyses used the same covariate-adjustment strategy. The corresponding results are provided in the Supplementary Materials.

#### 2.5.6. Analysis Hierarchy and Multiplicity Control

To address the issue of multiple comparisons, GDR-Total was designated as the primary dietary-quality outcome, whereas GDR-Health and GDR-Limit were considered secondary dietary-quality dimensions. The primary analysis evaluated the association between continuous MEQ score and GDR-Total using a fully adjusted multivariable linear regression model. Analyses involving GDR-Health and GDR-Limit were considered secondary analyses. Restricted cubic spline analyses, binary logistic regression contrasts, alternative knot specifications, bootstrap resampling, and tail-exclusion analyses were considered exploratory or sensitivity analyses.

The Benjamini–Hochberg false discovery rate procedure was applied to the *p* values from the primary linear regression analyses, and the corresponding FDR-adjusted *p* values were calculated accordingly. *p* values from secondary, exploratory, and sensitivity analyses were interpreted as nominal *p* values.

## 3. Results

### 3.1. Sample Characteristics

Demographic characteristics are presented in Table 1; the median age of participants was 15.5 years, with boys accounting for 52.4% (*n* = 515), and 66.3% (*n* = 651) residing in urban areas. Most participants reported a medium level of family economic status (74.6%), while 5.9% were from families with a low economic status. Regarding parental education, 70.9% of students’ fathers and 67.3% of students’ mothers had an educational attainment below the junior college level. In terms of physiological and behavioral characteristics, BMI classification showed that 78.3% of participants had a normal weight, while 15.3% were overweight and 6.4% were obese. Based on MEQ score, the majority were intermediate type (76.6%), followed by morning type (15.4%) and evening type (8.0%). Dietary quality also showed clear distribution patterns: 72.3% of students had a GDR-Total score ≥ 1 point; 87.3% had a GDR-Health score ≥ 3 points; and 73.7% had a GDR-Limit score ≥ 2 points.

### 3.2. Cross-Sectional Analysis of the Association Between Sleep Chronotype and Dietary Quality in a District of Xiamen

#### 3.2.1. Demographic Characteristics of Sleep Chronotypes

Univariate analysis results (Table 2) showed that there were significant differences in age, gender, GDR-Total score, GDR-Health score, grade, and residence among different MEQ chronotypes. In terms of age, the median ages of the morning, intermediate and evening types were 15, 15.5 and 15.5 years, respectively (*χ*^2^ = 6.248, *p* = 0.044). Regarding residence and gender, the proportion of intermediate type was higher among urban residents (76.5%), while the proportion of evening types was 4.8% among township residents, significantly lower than 9.7% among urban residents (*χ*^2^ = 9.426, *p* = 0.009). Boys exhibited a markedly higher prevalence of the morning chronotype (18.3%) compared to girls (12.2%) (*χ*^2^ = 9.648, *p* = 0.008). In grade distribution, the proportion of morning types was 21.2% and evening types 4.1% in junior high, whereas in senior high the proportions were 10.2% and 11.5%, respectively; the proportion of evening types was significantly higher in senior high than in junior high (*χ*^2^ = 36.502, *p* < 0.001). In the GDR-Total group, the proportion of evening types was highest in the GDR-Total < 0 group (18.1%) and lowest in the GDR-Total ≥ 1 group (6.2%), with a statistically significant between-group difference (*χ*^2^ = 23.353, *p* < 0.001). In the GDR-Health group, the proportion of evening-type was highest in the GDR-Health = 2 group (20.0%) and markedly lower in the GDR-Health ≥ 3 group (6.5%), with a similarly significant between-group difference (*χ*^2^ = 21.772, *p* < 0.001). In contrast, no statistically significant difference was observed across GDR-Limit group by MEQ chronotype (*χ*^2^ = 6.289, *p* = 0.179). This finding suggests that the GDR-Total score may be associated with sleep chronotypes.

#### 3.2.2. Distribution of Dietary Quality Across Demographic Characteristics in a District of Xiamen

Univariate analysis results (Table 3) showed that the distribution of GDR-Total score groups differed significantly by grade level, BMI, and sleep chronotypes. From the perspective of grade level, among junior high students, 76.4% had a GDR-Total score ≥ 1, which was substantially greater than the 68.7% observed in senior high students (*χ*^2^ = 7.655, *p* = 0.022). From the perspective of BMI, adolescents with overweight had the highest proportion of GDR-Total score ≥ 1 point (78.0%), followed by those with normal weight (72.3%) and obesity (58.7%). (*χ*^2^ = 12.705, *p* = 0.013). From the perspective of MEQ chronotype, among morning types, 77.5% had a GDR-Total score ≥ 1, which was significantly higher than the 55.7% of evening types and 73.0% of intermediate types (*χ*^2^ = 23.353, *p* < 0.001). These findings suggest that grade level, BMI, and sleep chronotypes may be important factors influencing the distribution of GDR-Total score and warrant further investigation.

#### 3.2.3. Multivariate Linear Regression Analysis of the Association Between Chronotype and Dietary Quality

**Figure 1:**
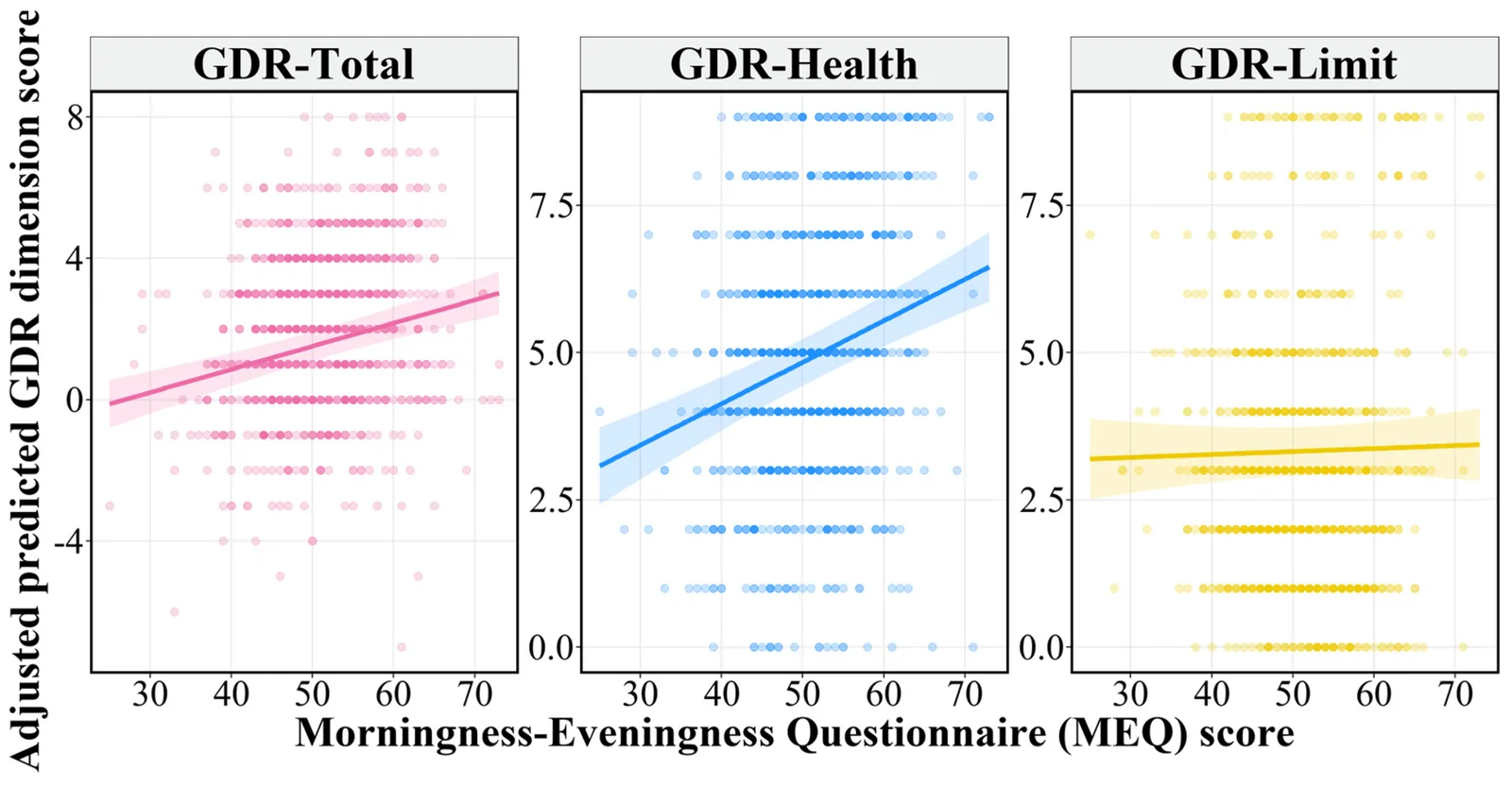
Covariate-adjusted linear associations between continuous MEQ scores and GDR dietary-quality dimensions. Points represent observed individual values. Solid lines represent adjusted predicted mean GDR dimension scores from multivariable linear regression models, and shaded bands indicate 95% confidence intervals. All models were adjusted for age, gender, BMI, grade, household income, residence, paternal education, maternal education, and sleep duration. Higher MEQ scores indicate a stronger morning preference. MEQ score was positively associated with GDR-Total and GDR-Health, whereas no statistically significant linear association was observed for GDR-Limit.

To examine the associations between chronotype and dietary quality, fully adjusted multivariable linear regression models were fitted for GDR-Health, GDR-Limit, and GDR-Total (Figure 1). MEQ score was positively associated with GDR-Health (*β* = 0.0707, 95% CI: 0.0507–0.0906; *p* < 0.001) and GDR-Total (*β* = 0.0662, 95% CI: 0.0455–0.0868; *p* < 0.001), whereas no statistically significant association was observed for GDR-Limit (*β* = 0.0045, 95% CI: −0.0163–0.0254; *p* = 0.670). The corresponding numerical results are presented in Table 4.

The fully adjusted models explained 7.0% of the variance in GDR-Total (R^2^ = 0.0698; adjusted R^2^ = 0.0534), 8.5% of the variance in GDR-Health (R^2^ = 0.0849; adjusted R^2^ = 0.0687), and 3.7% of the variance in GDR-Limit (R^2^ = 0.0366; adjusted R^2^ = 0.0196). Although the associations with GDR-Total and GDR-Health were statistically significant, their effect estimates were modest. These findings indicate positive associations of chronotype with the total and healthy dimensions of dietary quality, whereas evidence for an association with the limited-food dimension was weaker.

#### 3.2.4. Sensitivity Analysis of Associations Between MEQ Score and GDR Score

In sensitivity analyses, statistically significant associations were observed for selected GDR contrasts (Table 5). For GDR-Total < 0 versus ≥1, each 1-point increase in MEQ was associated with lower odds of this outcome (OR = 0.929, 95% CI: 0.901–0.957; *p* < 0.001). Compared with evening-type participants, morning-type and intermediate-type participants also had lower odds of GDR-Total < 0, with ORs of 0.179 (95% CI: 0.076–0.422; *p* < 0.001) and 0.342 (95% CI: 0.187–0.626; *p* < 0.001), respectively. For GDR-Health, each 1-point increase in MEQ was associated with lower odds of a score of 2 versus ≥3 (OR = 0.937, 95% CI: 0.900–0.975; *p* = 0.001). Consistent inverse associations were observed for both intermediate-type and morning-type participants in this contrast, with ORs of 0.305 (95% CI: 0.146–0.639; *p* = 0.002) and 0.266 (95% CI: 0.097–0.733; *p* = 0.01), respectively. For GDR-Limit, a 1-point increase in MEQ was associated with higher odds of a score of 0 versus ≥2 (OR = 1.060, 95% CI: 1.022–1.099; *p* < 0.001). This discrepancy between continuous and categorical results may imply that the influence of MEQ on the GDR-Limit follows a cumulative linear trend rather than a stepped categorical effect. It also suggests a potentially complex, non-linear relationship between these variables, warranting further exploration in future studies.

Compared with the previously reported findings, the dietary quality of adolescents in Xiamen appeared to be relatively favorable. To minimize potential bias arising from the original grouping of the GDR-Total score, we reclassified GDR-Total into three categories: ≤−2, −1 to 1, and ≥2 (Table S1). The reclassified analyses yielded similar results (Tables S2–S4).

### 3.3. Nonlinear Relationship Analysis Between MEQ Score and GDR Score

RCS analysis showed distinct nonlinear association patterns between MEQ score and GDR-Total, GDR-Health, and GDR-Limit. After adjustment for the prespecified covariates, statistically significant overall associations were observed for GDR-Total, GDR-Health, and GDR-Limit (all overall *p* < 0.001).

**Figure 2:**
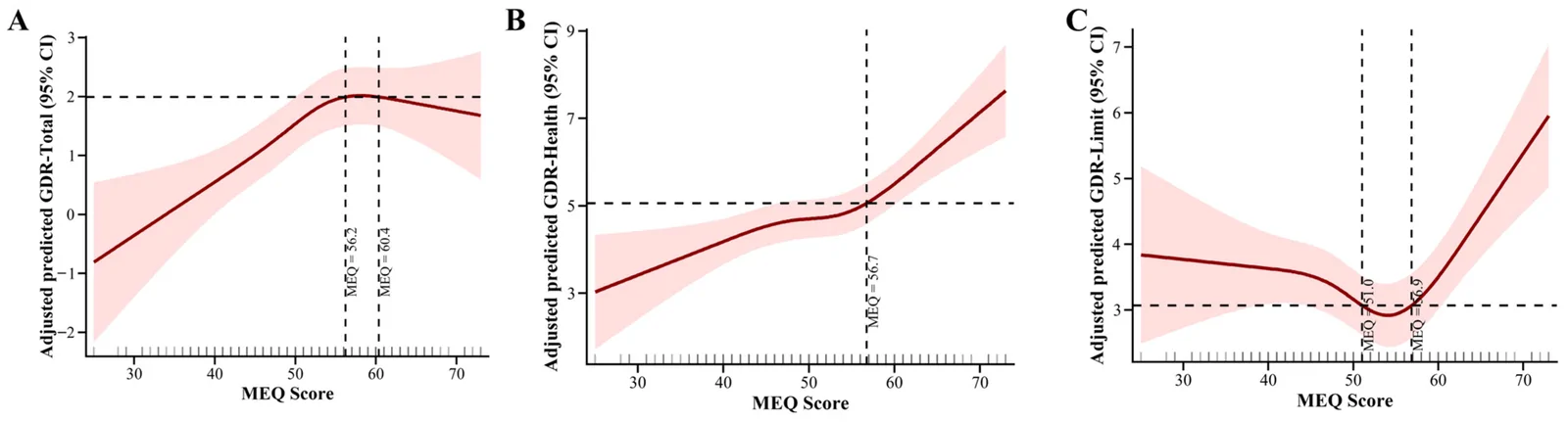
Covariate-adjusted restricted cubic spline associations between MEQ score and GDR dimensions. Panels show the associations for GDR-Total (**A**), GDR-Health (**B**), and GDR-Limit (**C**). Solid lines represent adjusted predicted mean GDR scores estimated using multivariable restricted cubic spline (RCS) models, and shaded bands indicate 95% confidence intervals. Rug marks along the x-axis show the distribution of observed MEQ scores. Dashed horizontal lines indicate the observed mean score for each GDR dimension, and dashed vertical lines indicate the MEQ values at which the fitted curves intersect the corresponding mean score. The primary models used four knots at MEQ scores of 40, 48, 54, and 63. Models were adjusted for age, gender, BMI, grade, household income, residence, paternal education, maternal education, and sleep duration. Higher MEQ scores indicate a stronger morning preference. The curves represent adjusted associations and should not be interpreted causally.

As shown in Figure 2A, GDR-Total increased with MEQ score at lower and intermediate levels, reached an apparent maximum at approximately MEQ = 58.0, and subsequently declined at higher MEQ scores. The nonlinear component was statistically significant (*p* = 0.014), indicating an apparent inverted-U-shaped association.

GDR-Health showed a predominantly increasing association across the observed MEQ range, although the nonlinear component was statistically significant (*p* = 0.019) (Figure 2B). The estimated maximum occurred at the upper boundary of the MEQ range, and no clear interior inverted-U pattern was observed. GDR-Limit showed a nonlinear U-shaped pattern, with a minimum at approximately MEQ = 53.9 followed by a marked increase at higher MEQ scores (*p* < 0.001) (Figure 2C). The shaded areas represent 95% confidence intervals, and the rug marks indicate the distribution of observed MEQ scores; the confidence intervals were wider near the extremes of the MEQ range.

The knot-sensitivity analysis showed similar apparent inverted-U curves for GDR-Total in the four- and five-knot models, whereas the three-knot model showed a predominantly increasing pattern without an interior maximum (Supplementary Figure S1A). The curves for GDR-Health remained predominantly increasing across all knot specifications (Supplementary Figure S1B). The U-shaped pattern for GDR-Limit was generally consistent across the three-, four-, and five-knot models (Supplementary Figure S1C). Overall, the apparent inverted-U pattern was specific to GDR-Total and should be interpreted cautiously because its precise shape varied according to the knot specification. These findings suggest that the associations between MEQ score and dietary quality dimensions may not be fully captured by simple linear models, particularly for GDR-Health and GDR-Limit. When individuals tend toward an extreme morning type, the GDR-Limit score increases markedly, indicating a higher intake of restricted foods. This finding raises the hypothesis that extreme morningness may be related to unmeasured factors associated with circadian misalignment.

Detailed results of the knot-sensitivity, bootstrap, and tail-exclusion analyses are presented in Supplementary Tables S5–S7 and Supplementary Figure S1.

## 4. Discussion

Sleep not only facilitates physiological restoration but also plays an important regulatory role in maintaining metabolic homeostasis [48,49,50]. The sleep–wake cycle and circadian rhythms play fundamental roles in energy metabolism and ingestive behaviors, directly influencing hunger, satiety, and food choices [51]. Individuals with delayed sleep phase or circadian misalignment tend to have higher BMI and face increased risks of chronic diseases, obesity, and other metabolic disorders [52,53]. In addition, both circadian misalignment and atypical chronotype patterns have been proposed as potential correlates of dietary behaviors, although these represent distinct constructs, thereby altering dietary structure and dietary quality [54]. During adolescence, intrinsic growth and development lead to a gradual delay in circadian rhythms. Meanwhile, external factors such as personal lifestyle, school schedules, and academic pressure make adolescents more susceptible to circadian disruption and its adverse metabolic consequences [55]. Therefore, this study primarily aimed to examine the association between chronotype and dietary quality among adolescents.

Our present study first identified a distinct developmental trend, with chronotypes gradually shifting from morning type to evening type as students progressed from junior to senior high school. Boys were more inclined to exhibit morning-type tendencies, a pattern consistent with the well-documented “phase delay” phenomenon during adolescence [16,56]. Additionally, significant differences in dietary behaviors were observed among adolescents with different sleep chronotypes; junior high school students had significantly better dietary quality than senior high school students, and morning-type and intermediate-type adolescents had better dietary quality than their evening-type peers.

Next, we employed multiple linear regression analysis to demonstrate that the MEQ score is significantly and positively associated with both GDR-Health and GDR-Total. This indicates that the improvement in overall dietary quality associated with morningness is primarily achieved through enhanced healthy dietary intake. Challenging the traditional view that a delayed sleep phase is linked to a higher intake of unhealthy food [57], a higher MEQ score was associated with higher GDR-Health and GDR-Total scores, although the magnitude of these associations was modest [58]. In contrast, a lower MEQ score suggests that the eveningness disadvantage is characterized by a deficiency in health-promoting dietary habits rather than a simple excess of dietary restrictions. Consequently, dietary interventions for evening-type adolescents should not only aim to reduce unhealthy food intake, but also emphasize increasing health-promoting food groups, such as breakfast foods, fruits, whole grains, and dairy products [59]. Accordingly, improving the intake of health-promoting foods may be a practical strategy for enhancing dietary quality and nutritional health among adolescents with different chronotypes.

Once again, a notable and potentially novel finding of this study was the identification of a potential nonlinear, apparently inverted-U-shaped association between MEQ score and GDR-Total score using RCS analysis. This result challenges the conventional linear assumption that “earlier is healthier” and provides a new hypothesis for future research. In the primary RCS model, predicted dietary quality initially increased as MEQ scores increased from eveningness toward morningness, reached a plateau, and subsequently declined at the extreme morningness end. The GDR-Limit subcomponent also showed a pronounced nonlinear trend. As MEQ scores increased into the extreme morningness range, GDR-Limit showed a steep increase, indicating greater consumption of foods recommended to be limited and a consequent potential decline in overall dietary quality, as reflected by GDR-Total. This phenomenon may be related to more rigid meal timing among extreme morning types or a mismatch between their biological rhythms and metabolic profiles and typical social eating schedules [60]. Therefore, the apparently inverted U-shaped association between MEQ score and GDR-Total may be driven primarily by the pronounced nonlinear variation in GDR-Limit. These findings suggest that a “circadian sweet spot” may exist for maintaining healthy dietary behaviors. Deviations from this optimal range, toward either eveningness or extreme morningness, may intensify the misalignment between endogenous biological rhythms and external social schedules, thereby compromising dietary quality. However, the apparent inverted-U pattern was sensitive to knot specification and observations at the distributional tails: the three-knot model did not identify an interior maximum, and the nonlinear component was no longer statistically significant after tail exclusion. This finding should therefore be interpreted as exploratory.

Taken together, these results challenge the simplistic view of morningness as an entirely beneficial factor and highlight the necessity of personalized nutritional interventions. Interventions should not adopt a uniform linear model, but should instead consider subtle individual differences across the chronotype spectrum. Specifically, individuals at both ends of the distribution, including extreme morning types and extreme evening types, may require different intervention strategies. Future precision-nutrition strategies may benefit from considering individual sleep–wake patterns and potential misalignment between biological and social schedules, including social jetlag.

Several limitations should be considered. First, the cross-sectional design precludes establishing temporal or causal relationships between chronotype and dietary quality. Second, the study was conducted in six randomly selected schools within one district of Xiamen, which may limit the generalizability of the findings to adolescents in other regions of China. Because school and class identifiers were not collected, school- or class-level clustering could not be explicitly accounted for in the analyses. Third, chronotype assessed using the MEQ, dietary quality assessed using the DQQ, and height and weight were all self-reported. Consequently, recall bias, social-desirability bias, and exposure or outcome misclassification, including potential BMI misclassification, cannot be excluded. Fourth, the DQQ primarily reflects dietary intake over the previous day or a relatively short period, whereas chronotype represents a more habitual individual preference. This mismatch in temporal frame may have attenuated or otherwise distorted the observed associations. Finally, physical activity, screen time, sleep quality, actual sleep timing, social jetlag, psychological distress, pubertal status, and objective circadian measures were not assessed. Although the analyses adjusted for all relevant covariates available in the dataset, residual confounding cannot be excluded. Therefore, the observed associations should not be interpreted as causal, and further longitudinal studies using objective measurements are warranted.

Looking forward, it is recommended to promote the application of validated instruments such as the MEQ-19 and DQQ for systematic screening of adolescent sleep chronotype and dietary quality. Moreover, interventions aimed at mitigating academic stress and other modifiable factors should be explored to optimize sleep chronotype profiles and thereby improve dietary behaviors, potentially offering novel avenues for addressing adolescent obesity and related health concerns. Tailored, integrative “routine and nutrition” intervention strategies should be developed to accommodate the heterogeneous health profiles of adolescents, ultimately enhancing their overall well-being and long-term health outcomes.

## 5. Conclusions

In conclusion, this study identified a potential nonlinear, apparently inverted U-shaped association between chronotype and dietary quality among adolescents in the primary model, challenging the assumption that greater morningness is uniformly beneficial. Although morning preference was generally associated with better overall dietary quality, this association was more evident for GDR-Health than for GDR-Limit. Both extreme eveningness and extreme morningness were associated with poorer dietary quality, suggesting a potentially optimal chronotype range for healthy eating patterns. These findings indicate that future interventions should consider sleep patterns alongside the promotion of health-enhancing food consumption, rather than focusing solely on restricting unhealthy foods.

## Figures and Tables

**Table 1:** Demographic characteristics.

Characteristics	Statistic
Age [M(P_25_, P_75_)]	15.5 (14.0, 17.0)
Gender (*n*, %)	
Boys	515 (52.4%)
Girls	467 (47.6%)
Grade (*n*, %)	
Junior high	462 (47.0%)
Senior high	520 (53.0%)
Residence (*n*, %)	
Town	331 (33.7%)
Urban	651 (66.3%)
Household income (*n*, %)	
High	10 (1.0%)
Upper middle	53 (5.4%)
Middle	733 (74.6%)
Lower middle	128 (13.1%)
Low	58 (5.9%)
Paternal education level (*n*, %)	
Junior high or below	345 (35.1%)
Senior high/vocational	352 (35.9%)
Junior college	170 (17.3%)
Bachelor’s degree or above	115 (11.7%)
Maternal education level (*n*, %)	
Junior high or below	381 (38.8%)
Senior high/vocational	280 (28.5%)
Junior college	223 (22.7%)
Bachelor’s degree or above	98 (10.0%)
BMI [M(P_25_, P_75_)]	20.2 (18.4, 22.9)
MEQ score [M(P_25_, P_75_)]	51 (46, 56)
GDR-Total score [M(P_25_, P_75_)]	2 (0, 4)
GDR-Health score [M(P_25_, P_75_)]	5 (4, 7)
GDR-Limit score [M(P_25_, P_75_)]	3 (1, 4)
BMI (*n*, %)	
Normal	769 (78.3%)
Overweight	150 (15.3%)
Obesity	63 (6.4%)
Chronotype (*n*, %)	
Morning type	151 (15.4%)
Intermediate type	752 (76.6%)
Evening type	79 (8.0%)
GDR-Total score group (*n*, %)	
<0 points	128 (13.0%)
=0 points	144 (14.7%)
≥1 point	710 (72.3%)
GDR-Health score group (*n*, %)	
≤1 point	55 (5.6%)
=2 points	70 (7.1%)
≥3 points	857 (87.3%)
GDR-Limit score group (*n*, %)	
=0 points	77 (7.8%)
=1 point	181 (18.4%)
≥2 points	724 (73.7%)

Note: M, median.

**Table 2:** Differences in chronotypes among demographic characteristics and eating behaviors.

Characteristic	Category	Morning Type n (%)/Median (P_25_, P_75_)	Intermediate Type n (%)/Median (P_25_, P_75_)	Evening Type n (%)/Median (P_25_, P_75_)	*χ* ^2^	*p*
Age	Median (P_25_, P_75_)	15.0 (13.5, 16.5)	15.5 (14.0, 17.0)	15.5 (14.0, 17.0)	6.248	**0.044**
Gender	Boys	94 (18.3)	388 (75.3)	33 (6.4)	9.648	**0.008**
	Girls	57 (12.2)	364 (77.9)	46 (9.9)		
Grade	Junior high	98 (21.2)	345 (74.7)	19 (4.1)	36.502	**<0.001**
	Senior high	53 (10.2)	407 (78.3)	60 (11.5)		
Residence	Town	61 (18.4)	254 (76.7)	16 (4.8)	9.426	**0.009**
	Urban	90 (13.8)	498 (76.5)	63 (9.7)		
Household income	High	3 (30.0)	5 (50.0)	2 (20.0)	9.900	0.272
	Upper middle	8 (15.1)	39 (73.6)	6 (11.3)		
	Middle	113 (15.4)	558 (76.1)	62 (8.5)		
	Lower middle	16 (12.5)	107 (83.6)	5 (3.9)		
	Low	11 (19.0)	43 (74.1)	4 (6.9)		
Paternal education level	Junior high or below	57 (16.5)	255 (73.9)	33 (9.6)	4.536	0.604
	Senior high/vocational	54 (15.3)	273 (77.6)	25 (7.1)		
	Junior college	22 (12.9)	138 (81.2)	10 (5.9)		
	Bachelor’s or above	18 (15.7)	86 (74.8)	11 (9.5)		
Maternal education level	Junior high or below	65 (17.0)	291 (76.4)	25 (6.6)	6.134	0.408
	Senior high/vocational	42 (15.0)	214 (76.4)	24 (8.6)		
	Junior college	32 (14.4)	174 (78.0)	17 (7.6)		
	Bachelor’s or above	12 (12.2)	73 (74.5)	13 (13.3)		
BMI	Normal	117 (15.2)	592 (77.0)	60 (7.8)	3.087	0.543
	Overweight	22 (14.7)	117 (78.0)	11 (7.3)		
	Obesity	12 (19.0)	43 (68.3)	8 (12.7)		
GDR-Total score group	<0 points	11 (8.6)	94 (73.4)	23 (18.1)	23.353	**<0.001**
	=0 points	23 (16.0)	109 (75.7)	12 (8.3)		
	≥1 point	117 (16.5)	549 (77.3)	44 (6.2)		
GDR-Health score group	≤1 point	6 (10.9)	40 (72.7)	9 (16.4)	21.772	**<0.001**
	=2 points	9 (12.9)	47 (67.1)	14 (20.0)		
	≥3 points	136 (15.9)	665 (77.6)	56 (6.5)		
GDR-Limit score group	=0 points	15 (19.5)	60 (77.9)	2 (2.6)	6.289	0.179
	=1 point	25 (13.8)	145 (80.1)	11 (6.1)		
	≥2 points	111 (15.3)	547 (75.6)	66 (9.1)		

Bold values indicate statistically significant differences (*p* < 0.05).

**Table 3:** Differences in GDR-Total score among demographic characteristics and chronotypes.

Characteristic	Category	<0 Points *n* (%)	=0 Points *n* (%)	≥1 Point *n* (%)	*χ* ^2^	*p*
Age	Median (P_25_, P_75_)	15.5 (14.0,16.0)	15.5 (14.0,16.75)	15.5 (14.0,17.0)	0.501	0.778
Gender	Boys	71 (13.8)	85 (16.5)	359 (69.7)	3.979	0.137
	Girls	57 (12.2)	59 (12.6)	351 (75.2)		
Grade	Junior high	49 (10.6)	60 (13.0)	353 (76.4)	7.655	**0.022**
	Senior high	79 (15.2)	84 (16.2)	357(68.7)		
Residence	Town	48 (14.5)	42 (12.7)	241 (72.8)	2.170	0.338
	Urban	80 (12.3)	102 (15.7)	469 (72.0)		
Household income	High	3 (30.0)	3 (30.0)	4 (40.0)	8.204	0.414
	Upper middle	10 (18.9)	7 (13.2)	36 (67.9)		
	Middle	95 (13.0)	105 (14.3)	533 (72.7)		
	Lower middle	13 (10.2)	19 (14.8)	96 (75.0)		
	Low	7 (12.1)	10 (17.2)	41 (70.7)		
Paternal education level	Junior high or below	49 (14.2)	43 (12.5)	253 (73.3)	8.959	0.176
	Senior high/vocational	47 (13.4)	59 (16.8)	246 (69.9)		
	Junior college	13 (7.6)	28 (16.5)	129 (75.9)		
	Bachelor’s or above	19 (16.5)	14 (12.2)	82 (71.3)		
Maternal education level	Junior high or below	53 (13.9)	54 (14.2)	274 (71.9)	12.388	0.054
	Senior high/vocational	33 (11.8)	46 (16.4)	201 (71.8)		
	Junior college	20 (9.0)	32 (14.3)	171 (76.7)		
	Bachelor’s or above	22 (22.4)	12 (12.2)	64 (65.3)		
BMI	Normal	103 (13.4)	110 (14.3)	556 (72.3)	12.705	**0.013**
	Overweight	17 (11.3)	16 (10.7)	117 (78.0)		
	Obesity	8 (12.7)	18 (28.6)	37 (58.7)		
Chronotypes	Morning type	11 (7.3)	23 (15.2)	117 (77.5)	23.353	**<0.001**
	Intermediate type	94 (12.5)	109 (14.5)	549 (73.0)		
	Evening type	23 (29.2)	12 (15.2)	44 (55.7)		

Bold values indicate statistically significant differences (*p* < 0.05).

**Table 5:** Sensitivity analysis of the association between MEQ scores and GDR dimensions.

MEQ Scores	GDR-Total			GDR-Health			GDR-Limit		
	Groups	OR (95%CI)	*p*	Groups	OR (95%CI)	*p*	Groups	OR (95%CI)	*p*
Continuous	<0 vs. ≥1	0.929 (0.901, 0.957)	**<0.001**	≤1 vs. ≥3	0.955 (0.916, 0.997)	**0.036**	=0 vs. ≥2	1.060 (1.022, 1.099)	**0.002**
	=0 vs. ≥1	0.988 (0.961, 1.016)	0.408	=2 vs. ≥3	0.937 (0.900, 0.975)	**0.001**	=1 vs. ≥2	1.012 (0.987, 1.038)	0.358
Categories									
Morning type	<0 vs. ≥1	0.179 (0.076, 0.422)	**<0.001**	≤1 vs. ≥3	0.278 (0.084, 0.919)	**0.036**	=0 vs. ≥2	3.868 (0.815, 18.361)	0.089
Intermediate type	<0 vs. ≥1	0.342 (0.187, 0.626)	**<0.001**	≤1 vs. ≥3	0.404 (0.175, 0.934)	**0.034**	=0 vs. ≥2	3.708 (0.862, 15.946)	0.078
Evening type	<0 vs. ≥1	Ref.		≤1 vs. ≥3	Ref.		=0 vs. ≥2	Ref.	
Morning type	=0 vs. ≥1	0.770 (0.338, 1.758)	0.535	=2 vs. ≥3	0.266 (0.097, 0.733)	**0.** **01**	=1 vs. ≥2	1.215 (0.533, 2.770)	0.643
Intermediate type	=0 vs. ≥1	0.775 (0.384, 1.566)	0.477	=2 vs. ≥3	0.305 (0.146, 0.639)	**0.002**	=1 vs. ≥2	1.534 (0.764, 3.082)	0.229
Evening type	=0 vs. ≥1	Ref.		=2 vs. ≥3	Ref.		=1 vs. ≥2	Ref.	

Notes: Covariates include age, gender, BMI, grade, household income, residence, parental education and sleep duration. CI, confidence interval; GDR, global dietary recommendations; OR, odds ratio; Ref, reference group. For each binary contrast, the first-listed outcome category was coded as 1 and the second-listed category as 0. Therefore, an OR > 1 indicates higher odds of the first-listed category, whereas an OR < 1 indicates lower odds. Separate binary logistic contrasts were used because the proportional-odds assumption for ordinal logistic regression was not satisfied. Bold values indicate statistically significant differences (*p* < 0.05).

**Table 4:** Fully adjusted linear regression analyses of the associations between MEQ scores and GDR dimensions.

Outcome	*β*	95% CI	*p* Value	FDR-Adjusted *p* Value	R^2^	Adjusted R^2^
GDR-Total	0.0662	[0.0455, 0.0868]	**<0.001**	**<0.001**	0.0698	0.0534
GDR-Health	0.0707	[0.0507, 0.0906]	**<0.001**	**<0.001**	0.0849	0.0687
GDR-Limit	0.0045	[−0.0163, 0.0254]	0.670	0.670	0.0366	0.0196

Notes: Models were adjusted for age, gender, BMI, grade, household income, residence, paternal education, maternal education, and sleep duration. *β* represents the adjusted mean change in the GDR score associated with a one-point increase in MEQ score. R^2^ and adjusted R^2^ represent the proportion of variance explained by the fully adjusted regression models. FDR adjustment was performed using the Benjamini–Hochberg method across the three prespecified linear regression tests. Bold values indicate statistically significant differences (*p* < 0.05).

## Data Availability

The datasets used and analyzed during the current study are available from the corresponding author on reasonable request.

## Supplementary Materials

The following supporting information can be downloaded at: https://www.mdpi.com/article/10.3390/nu18183097/s1, Supplementary Figure S1. Sensitivity analyses using alternative numbers of restricted cubic spline knots. Supplementary Table S1. Demographic characteristics. Supplementary Table S2. Differences of chronotypes among demographic characteristics and eating behaviors. Supplementary Table S3. Differences of GDR total score among demographic characteristics and chronotypes. Supplementary Table S4. Sensitivity analysis of the association between MEQ scores and GDR dimensions. Supplementary Table S5. Knot-sensitivity analyses for the association between MEQ score and GDR dimensions. Supplementary Table S6. Bootstrap stability analysis of the RCS curve patterns. Supplementary Table S7. Tail-exclusion sensitivity analysis.
