# Supplementary material for: Self-Reported Chronotype and Diet Quality in Chinese Adolescents: Insights from a Cross-Sectional Study in Xiamen
Source: Nutrients. 2026 Sep 21;18(18):3097. doi: 10.3390/nu18183097 (PMC13609943; doi:10.3390/nu18183097)
Supplement: Supplementary file 1 [file nutrients-18-03097-s001.zip › nutrients-4542003-supplementary.pdf]

Table S1 Demographic characteristics

Characteristics	Statistic
**Age [M(P_{25},P_{75})]**	15.5 (14.0,17.0)
**Gender (n, %)**	
Boys	515 (52.4%)
Girls	467 (47.6%)
**Grade (n, %)**	
Junior high	462 (47.0%)
Senior high	520 (53.0%)
**Residence (n, %)**	
Town	349 (35.5%)
Urban	633 (64.5%)
**Household income (n, %)**	
High	11 (1.1%)
Upper middle	55 (5.6%)
Middle	727 (74.1%)
Lower middle	131 (13.3%)
Low	58 (5.9%)
**Paternal education level (n, %)**	
Junior high or below	346 (35.2%)
Senior high/vocational	332 (33.8%)
Junior college	188 (19.1%)
Bachelor’s degree or above	116 (11.8%)
**Maternal education level (n, %)**	
Junior high or below	389 (39.6%)
Senior high / vocational	287 (29.2%)
Junior college	205 (20.9%)
Bachelor’s degree or above	101 (10.3%)
**BMI [M(P_{25},P_{75})]**	20.2 (18.2, 22.9)
**MEQ score [M(P_{25},P_{75})]**	51 (46, 56)
**GDR-Total score[M(P_{25},P_{75})]**	2 (0, 4)
**GDR-Health score [M(P_{25},P_{75})]**	5 (4, 7)
**GDR-Limit score [M(P_{25},P_{75})]**	3 (1, 4)
**BMI (n, %)**	
Normal	738 (75.2%)
Overweight	163 (16.6%)
Obesity	81 (8.2%)
**Chronotype (n, %)**	
Morning type	150 (15.3%)
Intermediate type	754 (76.8%)

Characteristics	Statistic
Evening type	78 (7.9%)
**GDR total score group (n, %)**	
≤ -2 points	52 (5.3%)
≤ 1, ≥ -1 points	362 (36.9%)
≥ 2 point	568 (57.8%)
**GDR-Health score group (n, %)**	
≤ 1 point	55 (5.6%)
= 2 points	69 (7.0%)
≥ 3 points	858 (87.4%)
**GDR-Limit score group (n, %)**	
= 0 points	77 (7.8%)
= 1 point	182 (18.6%)
≥ 2 points	723 (73.6%)

Note: M, median.

Table S2. Differences of chronotypes among demographic characteristics and eating behaviors

Characteristic	Category	Morning type n (%) / Median (P_{25}, P_{75})	Intermediate type n (%) / Median (P_{25}, P_{75})	Evening type n (%) / Median (P_{25}, P_{75})	χ^2	p
**Age**	Median (P_{25}, P_{75})	14.75 (13.0, 16.0)	15.5 (14.0, 17.0)	16 (15.0, 17.5)	31.666	**<0.001**
**Gender**	Boys	94 (18.3)	388 (75.3)	33 (6.4)	9.792	**0.007**
	Girls	56 (12.0)	366 (78.4)	45 (9.6)		
**Grade**	Junior high	97 (21.0)	346 (74.9)	19 (4.1)	35.215	**<0.001**
	Senior high	53 (10.2)	408 (78.5)	59 (11.3)		
**Residence**	Town	66 (18.9)	262 (75.1)	21 (6.0)	7.421	**0.024**
	Urban	84 (13.3)	492 (77.7)	57 (9.0)		
**Household income**	High	4 (36.3)	5 (45.5)	2 (18.2)	10.256	0.248
	Upper middle	7 (12.7)	42 (76.4)	6 (10.9)		
	Middle	110 (15.1)	558 (76.8)	59 (8.1)		
	Lower middle	17 (13.0)	107 (81.7)	7 (5.3)		
	Low	12 (20.7)	42 (72.4)	4 (6.9)		
**Paternal education level**	Junior high or below	58 (16.8)	255 (73.7)	33 (9.5)	4.615	0.594
	Senior high / vocational	49 (14.8)	260 (78.3)	23 (6.9)		
	Junior college	26 (13.8)	151 (80.3)	11 (5.9)		
	Bachelor's or above	17 (14.6)	88 (75.9)	11 (9.5)		
**Maternal education level**	Junior high or below	68 (17.5)	296 (76.1)	25 (6.4)	6.024	0.421
	Senior high / vocational	41 (14.3)	224 (78.0)	22 (7.7)		
	Junior college	29 (14.1)	157 (76.6)	19 (9.3)		
	Bachelor's or above	12 (11.9)	77 (76.2)	12 (11.9)		
**BMI**	Normal	114 (15.4)	568 (77.0)	56 (7.6)	2.135	0.711
	Overweight	21 (12.9)	126 (77.3)	16 (9.8)		
	Obesity	15 (18.5)	60 (74.1)	6 (7.4)		
**GDR total score group**	≤ -2 points	8 (15.4)	35 (67.3)	9 (17.3)	17.214	**0.002**
	≤ 1, ≥ -1 points	47 (13.0)	276 (76.2)	39 (10.8)		
	≥ 2 point	95 (16.7)	443 (78.0)	30 (5.3)		
**GDR-Health score group**	≤ 1 point	6 (10.9)	40 (72.7)	9 (16.4)	23.07	**<0.001**
	= 2 points	8 (11.6)	47 (68.1)	14 (20.3)		
	≥ 3 points	136 (15.9)	667 (77.7)	55 (6.4)		
**GDR-Limit score group**	= 0 points	14 (18.2)	61 (79.2)	2 (2.6)	5.765	0.217
	= 1 point	25 (13.7)	146 (80.2)	11 (6.0)		
	≥ 2 points	111 (15.4)	547 (75.7)	65 (9.0)		

Table S3. Differences of GDR total score among demographic characteristics and chronotypes

Characteristic	Category	≤ -2 points n (%)	≤ 1, ≥ -1 points n (%)	≥ 2 point n (%)	χ^2	p
**Age**	Median (P_{25},P_{75})	15.5 (14.25,16.5)	15.5 (14.0,17.0)	15.0 (14.0,17.0)	3.776	0.151
**Gender**	Boys	30 (5.8)	202 (39.2)	283 (55.0)	3.774	0.152
	Girls	22 (4.7)	160 (34.3)	285 (61.0)		
**Grade**	Junior high	20 (4.3)	150 (32.5)	292 (63.2)	10.450	**0.005**
	Senior high	32 (6.2)	212 (40.8)	276 (53.1)		
**Residence**	Town	17(4.9)	130 (37.2)	202 (57.9)	0.206	0.902
	Urban	35 (5.5)	232 (36.7)	366 (57.8)		
**Household income**	High	2 (18.2)	6 (54.5)	3 (27.3)	10.280	0.246
	Upper middle	5 (9.1)	22 (40.0)	28(50.9)		
	Middle	37 (5.1)	262 (36.0)	428 (58.9)		
	Lower middle	4 (3.1)	50 (38.2)	77 (58.8)		
	Low	4 (6.9)	22 (37.9)	32 (55.2)		
**Paternal education level**	Junior high or below	18 (5.2)	126 (36.4)	202 (58.4)	6.208	0.400
	Senior high / vocational	19 (5.7)	134 (40.4)	179 (53.9)		
	Junior college	8 (4.3)	58 (30.9)	122 (64.9)		
	Bachelor's or above	7 (6.0)	44 (37.9)	65 (56.0)		
**Maternal education level**	Junior high or below	21 (5.4)	143 (36.8)	225 (57.8)	6.867	0.333
	Senior high / vocational	15 (5.2)	109 (38.0)	163 (56.8)		
	Junior college	6 (2.9)	75 (36.6)	124 (60.5)		
	Bachelor's or above	10 (9.9)	35 (34.7)	56 (55.4)		
**BMI**	Normal	32 (4.3)	263 (35.7)	443 (60.0)	13.480	**0.009**
	Overweight	10 (6.1)	65 (39.9)	88 (54.0)		
	Obesity	10 (12.3)	34 (42.0)	37 (45.7)		
**Chronotype**	Morning type	8 (5.3)	47 (31.3)	95 (63.3)	17.214	**0.002**
	Intermediate type	35 (4.6)	276 (36.6)	443 (58.8)		
	Evening type	9 (11.5)	39 (50.0)	30 (38.5)		

Table S4. Sensitivity analysis of the association between MEQ scores and GDR dimensions

MEQ Scores	GDR total			GDR-Health			GDR-Limit		
	Groups	OR (95%CI)	p	Groups	OR (95%CI)	p	Groups	OR (95%CI)	p
Continuous	≤ -2 vs ≥ 2	0.945 (0.905, 0.987)	**0.011***	≤ 1 vs ≥ 3	0.963 (0.924, 1.003)	0.067	= 0 vs ≥ 2	1.065 (1.027, 1.104)	**0.001***
	≤1, ≥-1 vs ≥ 2	0.967 (0.947, 0.987)	**0.001***	= 2 vs ≥ 3	0.934 (0.899, 0.970)	**<0.001***	= 1 vs ≥ 2	1.015 (0.990, 1.040)	0.241
Categories									
Morning type	≤ -2 vs ≥ 2	0.287 (0.093, 0.884)	**0.031***	≤ 1 vs ≥ 3	0.319 (0.102–1.001)	0.050	= 0 vs ≥ 2	4.244 (0.905–19.903)	0.067
Intermediate type	≤ -2 vs ≥ 2	0.279 (0.116, 0.670)	**0.004***	≤ 1 vs ≥ 3	0.438 (0.195–0.982)	**0.045***	= 0 vs ≥ 2	4.005 (0.939–17.086)	0.061
Evening type	≤ -2 vs ≥ 2	Ref.		≤ 1 vs ≥ 3	Ref.		= 0 vs ≥ 2	Ref.	
Morning type	≤1, ≥-1 vs ≥ 2	0.375 (0.202–0.695)	**0.002***	= 2 vs ≥ 3	0.205 (0.075–0.558)	**0.002***	= 1 vs ≥ 2	1.338 (0.598–2.994)	0.479
Intermediate type	≤1, ≥-1 vs ≥ 2	0.478 (0.286–0.799)	**0.005***	= 2 vs ≥ 3	0.275 (0.137–0.553)	**<0.001***	= 1 vs ≥ 2	1.654 (0.834–3.283)	0.150
Evening type	≤1, ≥-1 vs ≥ 2	Ref.		= 2 vs ≥ 3	Ref.		= 1 vs ≥ 2	Ref.	

Notes：The first-listed outcome category was compared with the reference category. An OR >1 indicates higher odds of the first-listed category, whereas an OR <1 indicates lower odds. MEQ categories were compared with evening type as the reference group. All models were adjusted for age, gender, BMI, grade, household income, residence, paternal education, maternal education, and sleep duration.

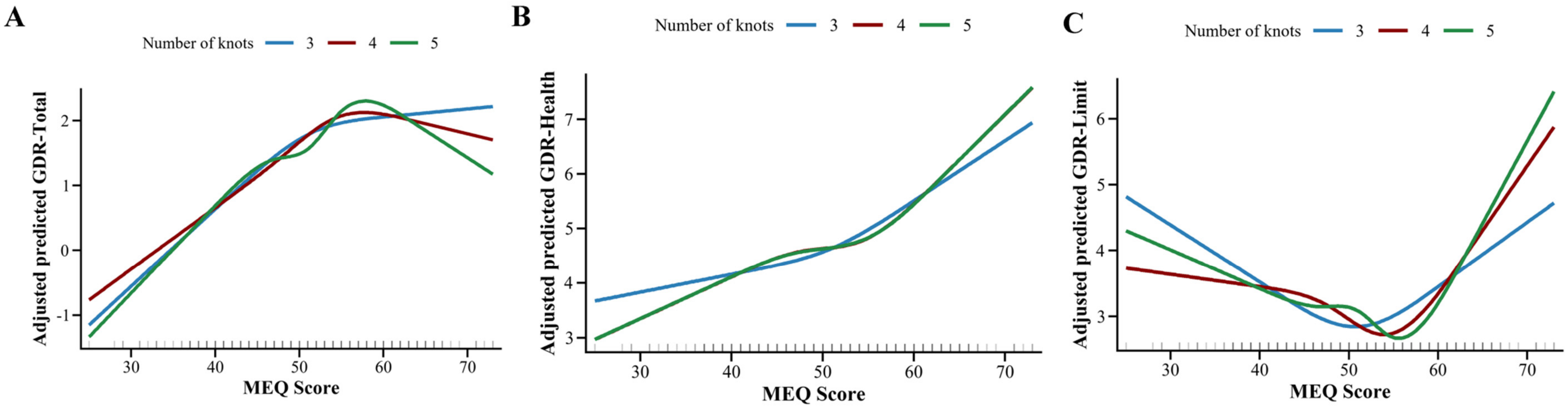

Supplementary Figure S1. Sensitivity analyses using alternative numbers of restricted cubic spline knots. Panels show the associations between MEQ score and GDR-Total (A), GDR-Health (B), and GDR-Limit (C). Colored lines represent covariate-adjusted predicted mean GDR scores from models using three knots (blue), four knots (red), or five knots (green). In panel B, the four- and five-knot curves largely overlapped across the lower-to-middle MEQ range and showed similar overall increasing patterns. The three-knot model used knots at the 10th, 50th, and 90th percentiles of MEQ (MEQ scores 42, 51, and 60); the four-knot model used knots at the 5th, 35th, 65th, and 95th percentiles (40, 48, 54, and 63); and the five-knot model used knots at the 5th, 27.5th, 50th, 72.5th, and 95th percentiles (40, 47, 51, 55, and 63). Rug marks along the x-axis indicate the distribution of observed MEQ scores. All models were adjusted for age, gender, BMI, grade, household income, residence, paternal education, maternal education, and sleep duration, categorized as ⩾7 hours versus <7 hours. This figure was intended to compare the stability of the fitted curve shapes across alternative knot specifications.

Table S5. Knot-sensitivity analyses for the association between MEQ score and GDR dimensions

Outcome	No. of knots	Percentiles	MEQ knot values	N	p overall	p nonlinear	Estimated maximum location
GDR-Total	3	10, 50, 90	42, 51, 60	982	<0.001	0.007	73.0 (upper boundary)
	4	5, 35, 65, 95	40, 48, 54, 63	982	<0.001	0.008	57.8 (interior)
	5	5, 27.5, 50, 72.5, 95	40, 47, 51, 55, 63	982	<0.001	0.004	57.8 (interior)
GDR-Health	3	10, 50, 90	42, 51, 60	982	<0.001	0.042	73.0 (upper boundary)
	4	5, 35, 65, 95	40, 48, 54, 63	982	<0.001	0.020	73.0 (upper boundary)
	5	5, 27.5, 50, 72.5, 95	40, 47, 51, 55, 63	982	<0.001	0.052	73.0 (upper boundary)
GDR-Limit	3	10, 50, 90	42, 51, 60	982	<0.001	<0.001	25.0 (lower boundary)
	4	5, 35, 65, 95	40, 48, 54, 63	982	<0.001	<0.001	73.0 (upper boundary)
	5	5, 27.5, 50, 72.5, 95	40, 47, 51, 55, 63	982	<0.001	<0.001	73.0 (upper boundary)

Notes: The 3-, 4-, and 5-knot models used the percentile definitions shown in the table. p overall compares the full RCS model with a covariate-only model. p nonlinear compares the full RCS model with a nested model containing a single linear MEQ term. Boundary locations do not represent an interior turning point. All models were adjusted for age, gender, BMI, grade, household income, residence, paternal education, maternal education, and sleep duration (≥7 hours vs <7 hours).

Table S6. Bootstrap stability analysis of the RCS curve patterns

Outcome	Original N	Bootstrap resamples	Apparent inverted-U pattern, %	Turning point: median; approximate 95% interval
GDR-Total	982	1000	71.4%	57.8; 55.2-73.0
GDR-Health	982	1000	0.0%	73.0 (upper boundary); 73.0-73.0
GDR-Limit	982	1000	0.0%	73.0 (upper boundary); 73.0-73.0

Notes: Bootstrap results were based on 1,000 nonparametric resamples of the primary four-knot model. The inverted-U proportion represents the percentage of valid resamples in which the fitted curve had an interior maximum with a positive left-hand slope and a negative right-hand slope. No inverted-U pattern was identified for GDR-Health or GDR-Limit.

Table S7. Tail-exclusion sensitivity analysis

Outcome	Lower MEQ threshold	Upper MEQ threshold	N after exclusion	p overall	p nonlinear	Estimated maximum location
GDR-Total	40	63	898	<0.001	0.996	63.0 (upper boundary)
GDR-Health	40	63	898	<0.001	0.067	63.0 (upper boundary)
GDR-Limit	40	63	898	0.107	0.089	63.0 (upper boundary)

Notes: Participants with MEQ scores below the 5th percentile or above the 95th percentile were excluded. The resulting restricted MEQ range was approximately 40-63. Boundary locations indicate that no interior maximum was identified after tail exclusion. Covariate adjustment was identical to that used in the primary RCS models.
